# MRI radiomics and nutritional-inflammatory biomarkers: a powerful combination for predicting progression-free survival in cervical cancer patients undergoing concurrent chemoradiotherapy

**DOI:** 10.1186/s40644-024-00789-2

**Published:** 2024-10-24

**Authors:** Qi Yan, Menghan- Wu, Jing Zhang, Jiayang- Yang, Guannan- Lv, Baojun- Qu, Yanping- Zhang, Xia Yan, Jianbo- Song

**Affiliations:** 1 Cancer Center, Tongji Shanxi Hospital, Shanxi Bethune Hospital, Shanxi Academy of Medical Sciences, Third Hospital of Shanxi Medical University, Taiyuan, Shanxi China; 2 Cancer Center, Shanxi Bethune Hospital, Third Hospital of Shanxi Medical University, Shanxi Academy of Medical Sciences Tongji Shanxi Hospital, Longcheng Street No.99, Taiyuan, China; 3 Shanxi Provincial Key Laboratory for Translational Nuclear Medicine and Precision Protection, Taiyuan, China; 4 China institute for radiation protection, Taiyuan, China; 5 Gynecological Tumor Treatment Center, the Second People’s Hospital of Datong, Cancer Hospital, Datong, China; 6 Imaging Department, the Second People’s Hospital of Datong, Cancer Hospital, Datong, China

**Keywords:** Cervical cancer, Concurrent chemoradiotherapy, MRI radiomics, Nutritional-inflammatory biomarkers, Prognosis

## Abstract

**Objective:**

This study aims to develop and validate a predictive model that integrates clinical features, MRI radiomics, and nutritional-inflammatory biomarkers to forecast progression-free survival (PFS) in cervical cancer (CC) patients undergoing concurrent chemoradiotherapy (CCRT). The goal is to identify high-risk patients and guide personalized treatment.

**Methods:**

We performed a retrospective analysis of 188 patients from two centers, divided into training (132) and validation (56) sets. Clinical data, systemic inflammatory markers, and immune-nutritional indices were collected. Radiomic features from three MRI sequences were extracted and selected for predictive value. We developed and evaluated five models incorporating clinical features, nutritional-inflammatory indicators, and radiomics using C-index. The best-performing model was used to create a nomogram, which was validated through ROC curves, calibration plots, and decision curve analysis (DCA).

**Results:**

Model 5, which integrates clinical features, Systemic Immune-Inflammation Index (SII), Prognostic Nutritional Index (PNI), and MRI radiomics, showed the highest performance. It achieved a C-index of 0.833 (95% CI: 0.792–0.874) in the training set and 0.789 (95% CI: 0.679–0.899) in the validation set. The nomogram derived from Model 5 effectively stratified patients into risk groups, with AUCs of 0.833, 0.941, and 0.973 for 1-year, 3-year, and 5-year PFS in the training set, and 0.812, 0.940, and 0.944 in the validation set.

**Conclusions:**

The integrated model combining clinical features, nutritional-inflammatory biomarkers, and radiomics offers a robust tool for predicting PFS in CC patients undergoing CCRT. The nomogram provides precise predictions, supporting its application in personalized patient management.

**Supplementary Information:**

The online version contains supplementary material available at 10.1186/s40644-024-00789-2.

## Introduction

Cervical cancer (CC) is the fourth most common malignancy among women worldwide [1]. According to the International Federation of Obstetrics and Gynaecology (FIGO) staging system, the stage at diagnosis significantly impacts patient prognosis. Patients diagnosed at an early stage can often achieve effective disease control and prolonged survival through surgical treatment [2]. However, due to the insidious nature and high malignancy of CC, many patients are diagnosed at an advanced stage, missing the optimal window for surgical intervention [3]. For these advanced-stage patients, concurrent chemoradiotherapy (CCRT) has become the primary treatment modality. Despite the significant improvements offered by CCRT, approximately 30% of patients still face the risk of recurrence and metastasis [4–8]. Current treatment options for recurrent or metastatic CC remain limited, leading to shorter survival times [9, 10]. Therefore, there is an urgent need to identify biomarkers that can predict survival risk, guide personalized treatment plans, and enhance clinical outcomes for these patients.

Imaging plays a pivotal role in monitoring tumor morphology and predicting patient outcomes. Magnetic Resonance Imaging (MRI) is the preferred modality for CC due to its superior tissue resolution, multiparametric capabilities, and multi-sequence imaging advantages. In particular, the combination of T2-weighted anatomical sequences with functional imaging techniques, such as Diffusion-Weighted Imaging (DWI), significantly enhances diagnostic accuracy for CC. DWI has become essential for evaluating lymph node metastasis, as standard MRI and dynamic contrast-enhanced MRI (DCE-MRI) offer no significant advantage over CT for lymph node staging. In addition, advent of multi-b value DWI further improves accuracy by generating fitted Apparent Diffusion Coefficient (ADC) values, which more precisely reflect tissue diffusion properties, aiding in the differentiation between benign and malignant lymph nodes [11, 12]. However, detecting small lesions or those that have not yet exhibited significant morphological changes can be challenging with the naked eye, potentially leading to delays in treatment for high-risk patients [13]. The emergence of radiomics has addressed this limitation. Through high-throughput and automated techniques, radiomics can extract a wide range of features from medical images and develop radiomic scores. These scores, when combined with regression models and support vector machines (SVM), facilitate quantitative analysis of images [14, 15]. The predictive power of radiomics based on MRI has been validated across various cancers, including CC [16–19].

Recent studies have increasingly recognized the critical role of inflammation response and nutritional deficiencies in tumor development and progression. These factors influence tumor growth through mechanisms such as inhibiting apoptosis, promoting angiogenesis, and inducing DNA damage [20, 21]. Tumor-induced inflammation is typically reflected in alterations in hematological parameters, including neutrophils, lymphocytes, monocytes, and platelets [22]. Systemic inflammatory markers, such as the neutrophil-to-lymphocyte ratio (NLR), platelet-to-lymphocyte ratio (PLR), SII, derived from peripheral blood, are valuable in assessing overall inflammation and stress levels in patients. These markers have been shown to predict adverse outcomes in cancer patients [22–25]. In addition, serum albumin is widely used as clinical indicator for evaluating nutritional status before treatment. However, newly developed immune-nutritional indices, such as the PNI and the Hemoglobin-Albumin-Lymphocyte-Platelet Index (HALP), combine albumin with various other parameters to provide a more comprehensive assessment of both nutritional status and immune function. These indices are increasingly being recognized and utilized by clinicians for a more nuanced understanding of patient health [26, 27].

Currently, composite models that combine radiomics, nutritional-inflammatory indices, and clinical features have shown superior prognostic value compared to models relying on single indicators alone [28–32]. For example, Liu et al. demonstrated that incorporating MRI radiomics into a composite model markedly enhanced its performance over models based solely on clinical data [19]. Similarly, Qu et al. showed that integrating the PNI, NLR, and other risk factors significantly improved the model’s predictive accuracy [33]. Despite these advancements, the combined use of clinical features, radiomics, and nutritional-inflammatory biomarkers for predictive purposes has not been thoroughly validated. This study aims to develop a predictive model that integrates clinical features, nutritional-inflammatory biomarkers, and radiomics to forecast 1-year, 3-year, and 5-year PFS probabilities in patients with CC undergoing CCRT.

## Materials and methods

### Patient selection

**Fig. 1:**
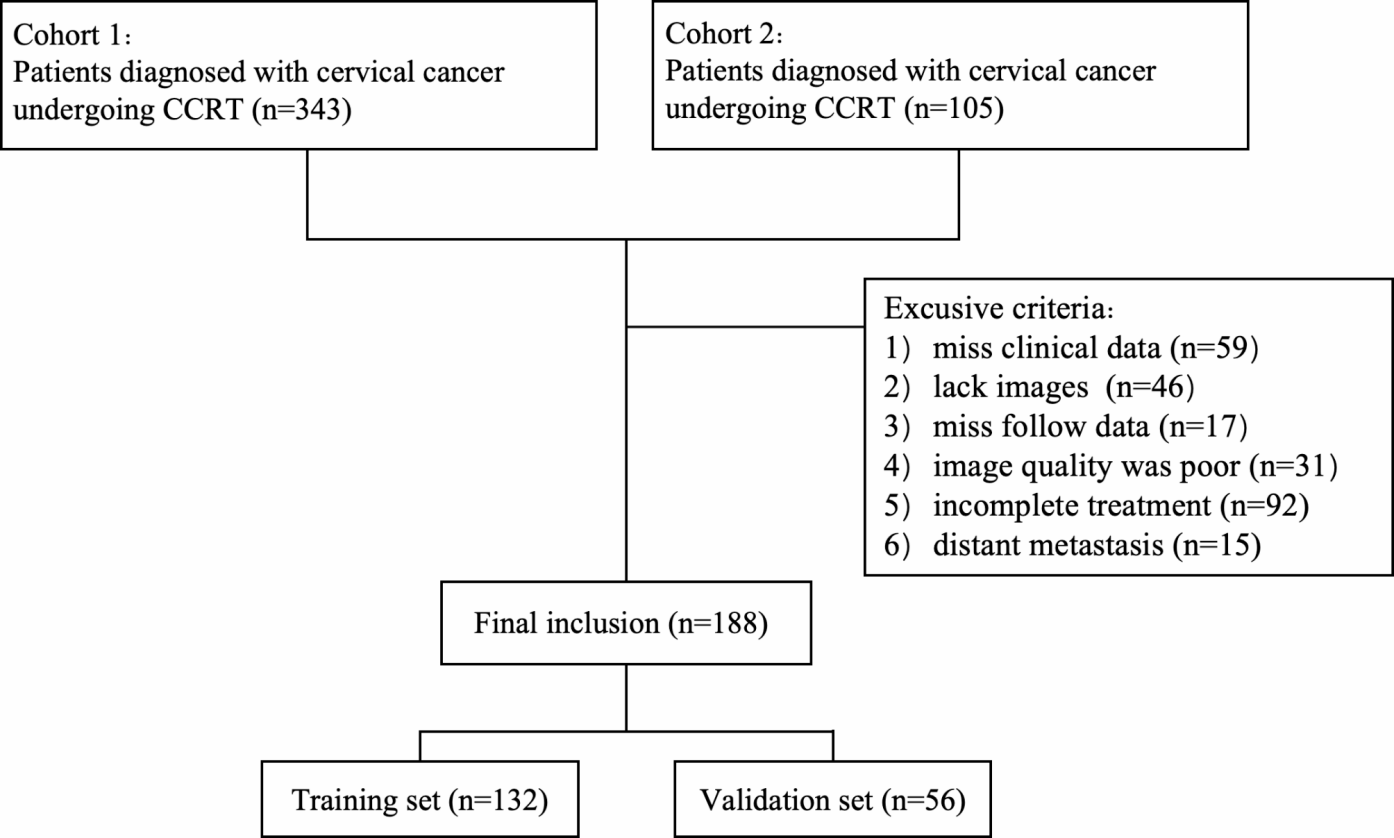
Flowchart of study population with inclusion and exclusion criteria

This study involved a retrospective analysis of patient cohorts from two independent hospitals: Shanxi Bethune Hospital (SBH, 2014–2022) and Shanxi Datong Second People’s Hospital Cancer Center (DSPHCH, 2020–2022). The inclusion criteria were as follows: (i) histologically confirmed squamous cell carcinoma of the cervix; (ii) FIGO stage IB to IVA based on the 2018 FIGO staging system; (iii) no prior surgical treatment; (iv) pelvic MRI conducted 1–2 weeks before treatment; (v) receipt of radical CCRT; (vi) complete hematological data available before treatment and within one week after external beam radiotherapy(EBRT) including white blood cell count (WBC, ×10^9/L), neutrophil count (NEU, ×10^9/L), absolute lymphocyte count (LYM, ×10^9/L), monocyte count (MONO, ×10^9/L), eosinophil(EOS, ×10^9/L ), hemoglobin (HB, g/L), and albumin (Alb, g/L). A total of 188 patients met these criteria, with 128 from SBH and 60 from DSPHCH. These patients were randomly assigned to a training set (132 patients) and a validation set (56 patients) in a 7:3 ratio. The flowchart of the study population is shown in Fig. 1.

Additionally, the study included clinical and pathological features with potential prognostic significance, such as age, disease stage (according to the 2008 FIGO staging system), and the presence of pelvic lymph node metastases.

### Nutritional-inflammatory biomarkers calculation

Peripheral blood indices were collected from patients before treatment (within 7 days prior to EBRT, referred to as “pre”) and after treatment (within 7 days following EBRT, referred to as “post”). The changes in biomarkers from pre- to post-treatment were calculated as follows (denoted as “change = post/pre”). The calculation of biomarkers was conducted as follows: (i) Changes in Systemic Inflammatory Markers: NLR___ change, PLR_ change, Monocyte-to-Lymphocyte Ratio (MLR_ change), Eosinophil-to-Lymphocyte Ratio (ELR_ change), SII_ change; (ii) Pre-Treatment Immune-Nutritional Indicators: HALP_ pre, PNI_ pre. Specific formulas for nutritional-inflammatory biomarkers are as follows: NLR = NEU / LYM; PLR = PLT / LYM, MLR = MONO / LYM; SII = PLT*NEU / LYM; ENLR = EOS / LYM; PNI = Alb ( g / L ) + 5*LYM; HALP = Hb*Alb*LYM / PLT.

### MRI protocol and tumor segmentation

All MRI images were acquired using a 3.0 T clinical MRI scanner (GE Signa HDXT 3.0T MRI, GE Healthcare, USA) with a phased-array 8-channel abdominal coil. Patients were positioned supine and instructed to hold their breath during scanning. The imaging range covered the entire pelvis, from the upper margin of the iliac crests to the lower margin of the pubic symphysis. The imaging sequences included axial T2-weighted (T2WI_ax), sagittal T1-weighted (T1WI_sag), sagittal T2-weighted (T2WI_sag), coronal T2-weighted (T2WI_cor), axial diffusion-weighted imaging (DWI) with b-values of 0 s/mm² and 800 s/mm², and apparent diffusion coefficient (ADC) maps. Detailed parameters for each sequence are provided in S1.

T2WI_ax, T2WI_sag, and DWI images were retrieved from the Picture Archiving and Communication System (PACS, Carestream, Canada) and exported in DICOM format. Manual tumor segmentation was performed using ITK-SNAP software (v.3.6, www.itksnap.org). A radiologist with 20 years of experience in pelvic MRI (Reader 1) manually delineated the tumor regions of interest (ROIs) on a slice-by-slice basis, carefully avoiding areas of cystic degeneration, necrosis, or hemorrhage. After segmentation, all tumor layers were exported in NIFTI format to create a 3D volume of interest (VOI). To assess inter-observer reproducibility, a second round of tumor segmentation was conducted on 50 randomly selected patients by a radiologist with 10 years of experience in gynecological imaging (Reader 2).

### Radiomics feature extraction and selection

Before feature extraction, image preprocessing was conducted to reduce variability introduced by different MRI scanners. All images underwent Z-score normalization to ensure a standard normal distribution with a mean of 0 and a standard deviation of 1. The images were then resampled to a voxel size of 1 × 1 × 1 mm. Radiomics features were extracted using the open-source Python-based tool Pyradiomics. The extracted features included: (i) First-order statistics; (ii) Shape features; (iii) Texture features: Gray-Level Co-occurrence Matrix (GLCM), Gray-Level Size Zone Matrix (GLSZM), Gray-Level Run Length Matrix (GLRLM), Neighboring Gray Tone Difference Matrix (NGTDM), Gray-Level Dependence Matrix (GLDM); (iv)Higher-order features: Wavelet features, Laplacian of Gaussian (LoG) features, Gradient features, Local Binary Pattern (LBP) features.

**Fig. 2:**
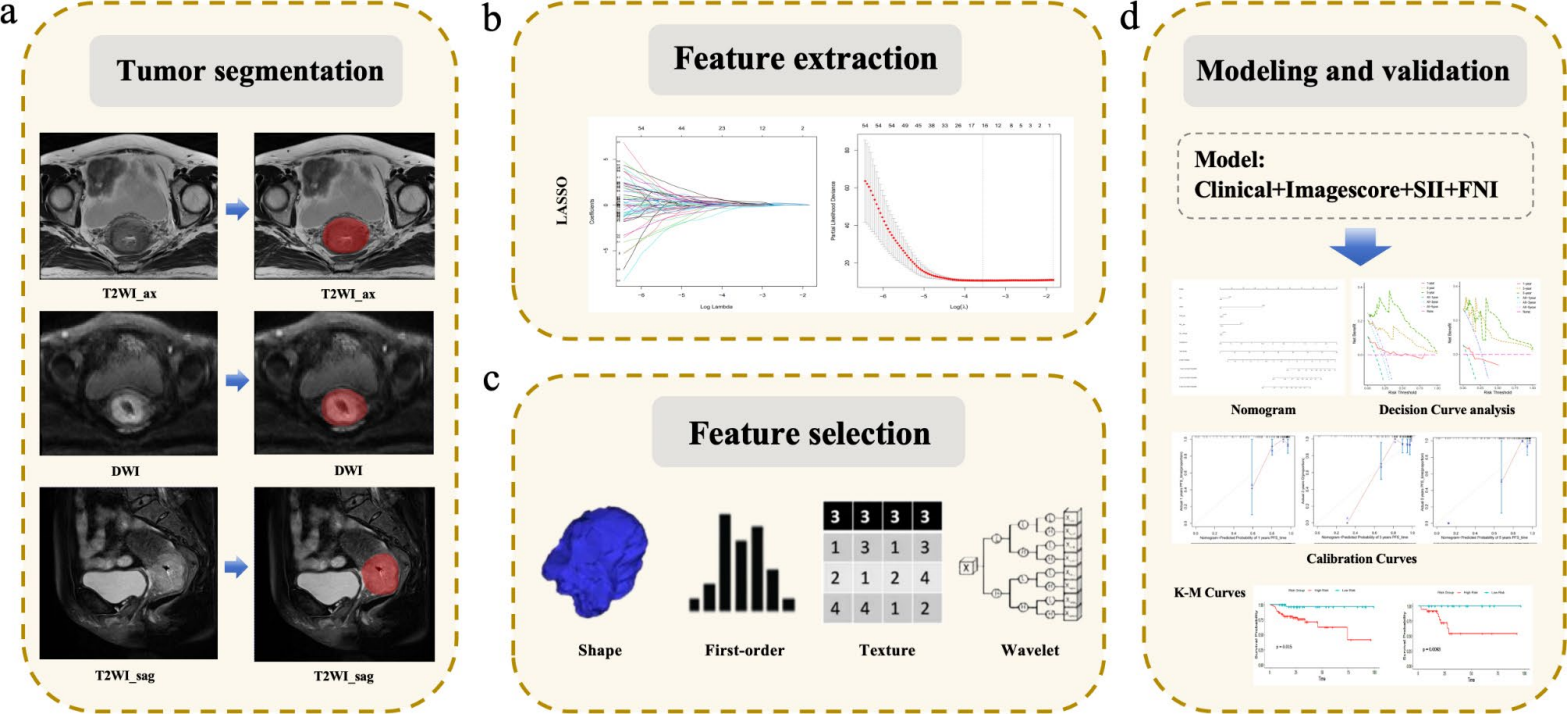
Radiomics flow chart for predicting PFS in patients with cervical cancer

To evaluate feature reproducibility, the Intraclass Correlation Coefficient (ICC) was used, retaining only features with an ICC greater than 0.9. Univariate analysis was then performed to identify features with significant differences (*P* < 0.05). Pearson correlation analysis was used to remove redundant features. Finally, the Least Absolute Shrinkage and Selection Operator (LASSO) regression method was employed to select the final set of features. The radiomics score (ImageScore) was calculated using the formula: ImageScore = coefficient 1 × feature 1 + coefficient 2 × feature 2 + …. The workflow for radiomics feature extraction and selection is illustrated in Fig. 2.

### Model and nomogram development and evaluation

We constructed five combined models:

i) **Model 1: Clinical and Radiomics Model**. This model was built using clinical features and radiomics data.
ii) **Model 2: Clinical and Nutritional-Inflammatory Biomarkers Model.** This model was built using clinical features, systemic inflammatory markers, and immune-nutritional biomarkers.
iii) **Model 3: Clinical**,** Systemic Inflammatory Markers**,** and Radiomics Model**. This model was built using clinical features, systemic inflammatory markers, and radiomics data.
iv) **Model 4: Clinical**,** Immune-Nutritional Biomarkers**,** and Radiomics Model**. This model was built using clinical features, immune-nutritional biomarkers, and radiomics data.
v) **Model 5: Clinical**,** Nutritional-Inflammatory Biomarkers**,** and Radiomics Model**. This model was built using clinical features, systemic inflammatory markers, immune-nutritional biomarkers, and radiomics data.

To evaluate the predictive performance of these models in both the training and validation sets, we calculated the C-index and its 95% confidence interval (CI) for each model. The optimal model was used to develop a prognostic nomogram for PFS, mapping actual scores to corresponding point coordinates. The nomogram’s predictive ability was validated by plotting ROC curves for 1-year, 3-year, and 5-year PFS. Calibration curves were generated to demonstrate the agreement between predicted and actual outcomes. Finally, the clinical utility of the nomogram was evaluated by DCA.

### Treatment and follow-up

All patients underwent EBRT combined with intracavitary brachytherapy (ICBT) and concurrent platinum-based chemotherapy. EBRT was administered at a total dose of 45.0–50.4 Gy in daily fractions of 1.8–2.0 Gy, over 5–6 weeks, with treatment given 5 times per week. ICBT was initiated between the 15th EBRT session and the end of EBRT, or within one week after EBRT completion, with a total dose of 30–45 Gy, administered in 5–6 Gy fractions. Concurrent chemotherapy, typically with cisplatin or nedaplatin at a dose of 30–40 mg/m², was started during the first week of radiotherapy.

PFS was the primary endpoint, defined as the time from the start of CCRT to disease progression, death, or the last follow-up (censoring date: August 31, 2023). Follow-up visits were conducted every 3–6 months for the first 2 years post-CCRT, every 6–12 months during years 3–5, and annually thereafter.

### Statistical analysis

Statistical analyses were performed using SPSS version 26.0 and R version 4.1.2. The methods used were: (i) Baseline comparison of patients’ characteristics: Categorical variables were presented as n(%) and compared using chi-square tests or Fisher’s exact tests; (ii) Optimal cutoff values for continuous variables were determined using X-tile software, converting them into binary variables. (iii) Radiomics features were selected using LASSO-Cox regression, and regression coefficients were calculated to derive the radiomics score (ImageScore); (iv) Risk scores calculated by nomogram and ImageScore were used to classify patients into high-risk and low-risk groups based on the cutoff values. Kaplan-Meier (K-M) survival curves and log-rank tests were used to compare PFS between the two groups; (v) Cox proportional hazards regression analysis was conducted to identify independent prognostic factors for PFS in CC. All statistical tests were two-sided, with a significance level set at *P* < 0.05.

## Results

### Patient characteristics

Baseline characteristics showed no significant clinical differences between the training and validation sets in terms of clinical features, systemic inflammatory markers, and immune-nutritional indicators (Table 1; all *P* > 0.05). The median follow-up duration for the entire cohort was 31.40 months, with a range of 8.97 to 99.50 months. At the final follow-up, disease progression was observed in 24 patients (18.2%) in the training set and 11 patients (30.8%) in the validation set.

**Table 1:** Clinical and pathological characteristics of patients in the training and validation cohorts

	Total (*N*= 188) No.(%)	Training cohort (*n*=132) No.(%)	Validation cohort (*n*=56) No.(%)	*P* value
Age				0.87
<62	89(47.3%)	63(47.7%)	26(46.4%)	
≥62	99(52.7%)	69(52.3%)	30(53.6%)	
Stage				0.71
I-II	94(50.0%)	66(50.0%)	28(50.0%)	
III-IV	94(50.0%)	66(50.0%)	28(50.0%)	
SCC_Ag				0.21
<27.0	140(74.5%)	102(77.3%)	38(67.9%)	
≥27.0	48(25.5%)	30(22.7%)	18(32.1%)	
Pelvic LNM				0.16
Yes	81(43.1%)	61(46.2%)	20(35.7%)	
No	107(56.9%)	71(53.8%)	36(64.3%)	
NLR_ change				0.62
<0.4	73(38.8%)	56(42.4%)	17(30.3%)	
≥0.4	115(61.2%)	76(57.6%)	39(69.6%)	
PLR_ change				0.99
<2.1	147(78.2%)	105(79.5%)	42(75.0%)	
≥2.1	41(21.8%)	27(20.4%)	14(25.0%)	
MLR_ change				0.11
<0.3	45(23.9%)	32(24.2%)	13(23.2%)	
≥0.3	143(76.1%)	100(75.8%)	43(76.8%)	
SII_ change				0.50
<2.6	118(62.8%)	82(62.1%)	36(64.3%)	
≥2.6	70(37.2%)	50(37.9%)	20(35.7%)	
ELR_ change				0.68
<0.1	35( 18.6%)	27(20.5%)	8( 14.3%)	
≥0.1	153(81.4%)	105(79.5%)	48(85.7%)	
HALP_ pre				0.99
<30.8	82(43.6%)	57(43.2%)	25(44.6%)	
≥30.8	106(56.4%)	75(56.8%)	31(55.4%)	
FNI_ pre				0.99
<44.7	91(48.4%)	53(47.0%)	29(51.8%)	
≥44.7	97((51.6%)	79(53.0%)	27(48.2%)	
Clinical endpoints				0.11
None	153(81.4%)	108(81.8%)	45(69.2%)	
Recurrence or distant metastasis	35( 18.6%)	24( 18.2%)	11(30.8%)	

SCC-Ag=squamous cell carcinoma antigen; pre=pre-concurrent radiochemotherapy; change=post/pre concurrent radiochemotherapy; NLR=neutrophil-to-lymphocyte ratio; PLR=platelet-to-lymphocyte ratio; MLR=monocyte to lymphocyte ratio; SII=systemic immunoinflammatory index; PNI=prognostic nutritional index; HALP=hemoglobin, albumin, lymphocyte, platelet; ELR=eosinophil-to-lymphocyte ratio

Univariate Cox regression analysis revealed several factors significantly associated with PFS. Among clinical features, age (HR = 0.479, 95% CI: 0.235–1.023, *P* = 0.479), SCC-Ag (HR = 3.387, 95% CI: 1.545–7.354, *P* = 0.0023), and stage (HR = 5.180, 95% CI: 2.240–11.950, *P* < 0.0001) were associated with PFS. In systemic inflammatory markers, SII (HR = 2.513, 95% CI: 1.082–5.832, *P* = 0.0323) was significantly associated with PFS. Among immune-nutritional indicators, FNI (HR = 0.335, 95% CI: 0.172–0.649, *P* = 0.0013) was also significantly associated with PFS (S2).

### Feature selection and imagescore construction

1,153 radiomic features were extracted from each imaging sequence: T2WI_ax, T2WI_sag, and DWI, within the VOI. After a comprehensive multi-step selection process, 16 features were identified as particularly valuable. The ImageScore, calculated using these features, was found to be an independent prognostic factor for PFS in CC (HR = 2.358; 95% CI: 1.480–3.758). Detailed information on the selected features and their coefficients is provided in S3.

**Fig. 3:**
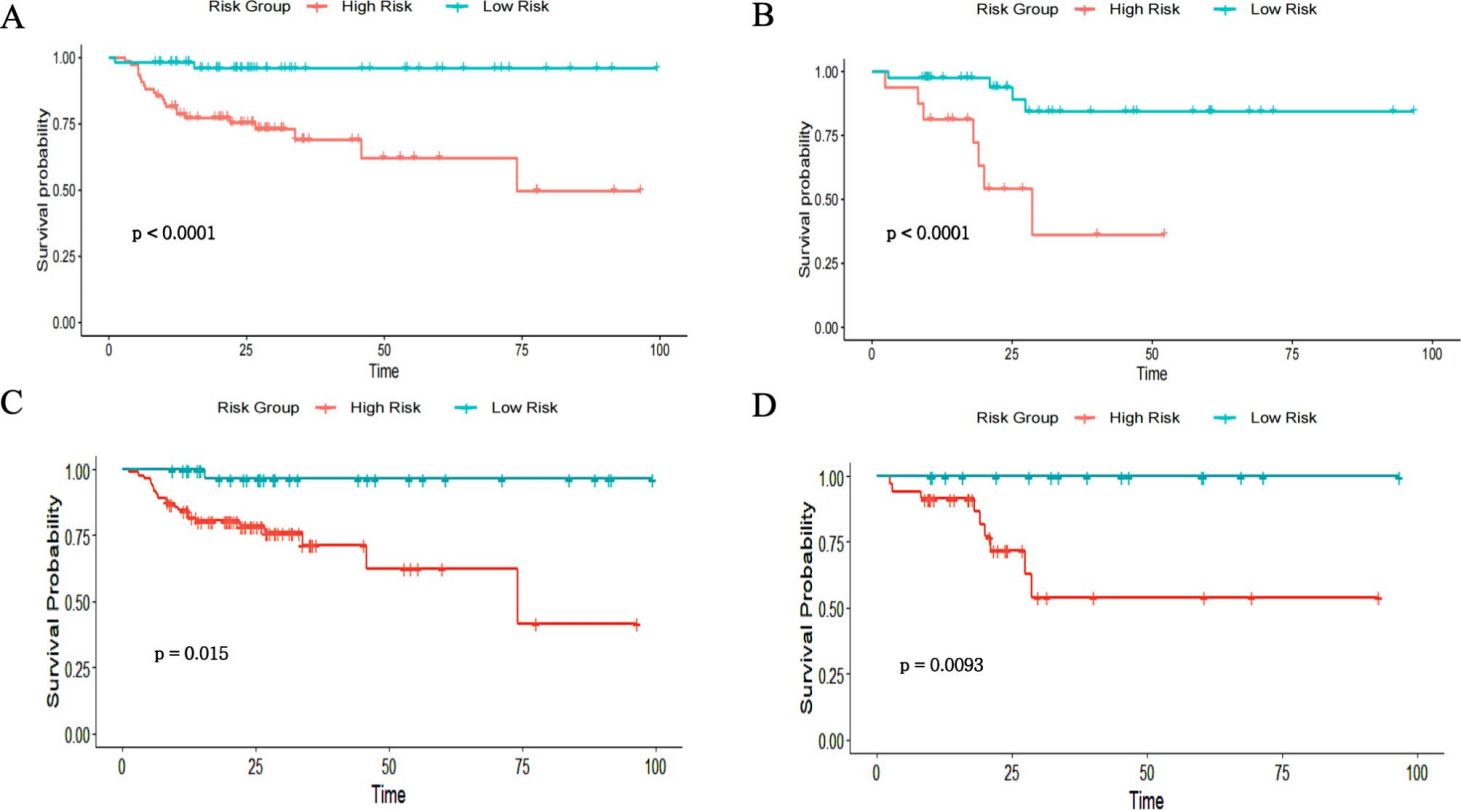
The Kaplan–Meier curves for survival in CCRT CC stratified in various risk stratification subgroups in the training cohort and validation cohort. Patients with high and low risks of PFS were stratified by the nomogram in the training (**A**) and validation (**B**) cohorts. Patients with high and low risks of PFS were stratified by the ImageScore in the training (**C**) and validation (**D**) cohorts. The log-rank test was used to calculate P values

Patients were categorized into high-risk and low-risk groups using an ImageScore cutoff of -0.34. K-M curves revealed significant survival differences between these groups in both the training set (Fig. 3A) and validation set (Fig. 3B), with the high-risk group showing markedly lower PFS compared to the low-risk group (both *P* < 0.0001, log-rank test).

### Multiple models construction and evaluation

In constructing and evaluating multivariable Cox proportional hazards models incorporating clinical features (such as age, stage, and SCC-Ag), SII, FNI, and ImageScore, we found that, after adjusting for various auxiliary factors, only stage and ImageScore maintained significant relevance to PFS (see S2). Based on Cox univariate analysis, we developed the following models: Model 1 includes clinical features and ImageScore; Model 2 integrates clinical features with SII_ change and FNI_ pre; Model 3 combines clinical features with SII_ change and ImageScore; Model 4 merges clinical features with FNI_ pre and ImageScore; and Model 5 encompasses clinical features, SII_ change, FNI_ pre, and ImageScore.

As detailed in Table 2, in the training set, all models except the one incorporating clinical features and nutritional-inflammation biomarkers outperformed the model based on stage and radiomics from Cox multivariate analysis. Among the five models, Model 5 (C-index: 0.833; 95% CI: 0.792–0.874) and Model 4 (C-index: 0.833; 95% CI: 0.793–0.873) demonstrated similar performance in predicting PFS following CCRT in CC patients. However, in the validation set, Model 5 achieved superior predictive performance compared to the other models (C-index: 0.789; 95% CI: 0.679–0.899). Therefore, based on its predictive accuracy and validation results, Model 5, which incorporates clinical features, SII_ change, FNI_ pre, and ImageScore, is identified as the optimal model.

**Table 2:** C-indexes of six combined models

Models	Training cohort(*n*=132)	Validation cohort(*n*=56)
Stage+Imagescore	0.822(95%CI: 0.781-0.863)	0.785(95%CI: 0.653-0.917)
Clinical+Imagescore	0.826(95%CI: 0.785-0.867)	0.781(95%CI: 0.652-0.920)
Clinical+FNI+SII	0.778(95%CI: 0.727-0.829)	0.784(95%CI: 0.673-0.895)
Clinical+Imagescore+FNI	0.826(95%CI: 0.783-0.869)	0.783(95%CI: 0.674-0.892)
Clinical+Imagescore+SII	0.833(95%CI: 0.793-0.873)	0.776(95%CI: 0.646-0.906)
Clinical+Imagescore+FNI+SII	0.833(95%CI: 0.792-0.874)	0.789(95%CI: 0.679-0.899)

Clinical data included age, stage, SCC_Ag; The Imagescore means radiomics score; CI=confidence interval; SII=systemic immunoinflammatory index; PNI=prognostic nutritional index

### Nomogram development and validation

**Fig. 4:**
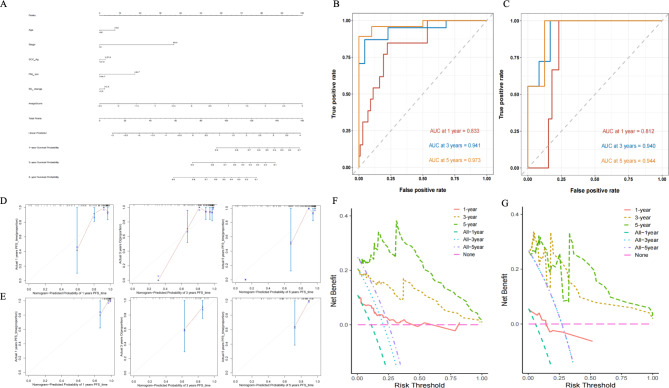
Nomogram and its prediction efficacy for CC patients receiving CCRT. Nomogram predicting the one-, three- and five-year PFS (**A**). The ROC curve of the nomogram for the one-, three- and five-year PFS in the training (**B**) and validation cohorts (**C**). Calibration curves of nomogram for the one-, three- and five-year PFS in the training (**D**) and validation cohorts (**E**). Decision curve analysis of the nomogram for the one-, three- and five-year PFS in the training (**F**) and validation cohorts (**G**). In the calibration curve, predicted survival probabilities by the nomogram are plotted on the x-axis, while actual survival rates are plotted on the y-axis. The diagonal dashed line represents perfect prediction. Solid blue lines depict the performance of the nomogram at 1 year, 3 years, and 5 years, respectively, with closer proximity to the dashed line indicating higher accuracy

Our results demonstrate that Model 5 exhibits superior predictive performance in both the training and validation sets. Therefore, we converted Model 5 into a nomogram to predict 1-year, 3-year, and 5-year PFS (Fig. 4A). This nomogram visualizes each patient’s risk factors and predicted outcomes, allowing us to aggregate scores and compute the probabilities of 1-year, 3-year, and 5-year survival using the total point axis.

The ROC curves for the 1-year, 3-year, and 5-year PFS, as derived from the nomogram, show an AUC of 0.833, 0.941, and 0.973 in the training set, respectively, and 0.784, 0.940, and 0.944 in the validation set (Fig. 4B and C). Calibration curves (Fig. 4D and E) reveal excellent agreement between observed outcomes and nomogram predictions, confirming the nomogram’s high accuracy in forecasting PFS for CC patients after CCRT. Furthermore, DCA (Fig. 4F and G) indicates that the nomogram provides significant net benefits in predicting PFS at 3 years and 5 years, with the greatest net benefit observed for 5-year PFS, emphasizing its clinical utility.

The total points were mapped to the linear predictor in the nomogram to differentiate patient risk levels according to the cutoff (0.13). K-M curves indicated that the high-risk group had significantly shorter PFS compared to the low-risk group in both the training set (Fig. 3C) and the validation set (Fig. 3D), highlighting a poorer survival outcome for high-risk patients.

## Discussion

This study investigates the predictive value of integrating clinical features, systemic inflammatory markers, immune-nutritional indices, and radiomic features for PFS in patients with CC undergoing CCRT. Our findings demonstrate that a combined prognostic model incorporating clinical characteristics (age, stage, and SCC-Ag), SII, FNI, and radiomic features provides superior predictive performance, achieving the highest C-index among all evaluated models. Additionally, we developed a nomogram based on this integrated model to predict 1-year, 3-year, and 5-year PFS for CC patients. The nomogram has been validated for accuracy and clinical utility, effectively stratifying patients into high-risk and low-risk groups and showing clear correlations between risk scores and survival outcomes. These results were supported by validation set data, confirming the model’s robustness and practical relevance.

Clinical features such as age, stage, LNM, and tumor markers are traditionally used to assess treatment response and prognosis in CC [34–37]. Our study reaffirms the significant roles of stage, age, and SCC-Ag in predicting PFS. However, we observed a weaker association between LNM and PFS. This discrepancy may be due to our focus on the presence of pelvic lymph node metastasis alone, without considering the size, shape, number of metastatic lymph nodes, or metastasis in other regions. The omission of these factors might have influenced the prognostic value of LNM.

In clinical practice, it is common to observe significant variations in survival outcomes among patients with similar clinical characteristics. This underscores the urgent need for objective and efficient assessment methods to address this challenge. Radiomics, which involves analyzing the entire tumor and extracting high-dimensional features, offers a more comprehensive prognostic evaluation than relying on individual clinical factors alone. Numerous studies have highlighted the substantial advantages of radiomics in cancer prognosis assessment [38]. Specifically, MRI, with its excellent soft tissue resolution and multi-sequence imaging capabilities, has demonstrated considerable success in predicting survival outcomes in CC. While early research primarily focused on extracting features from single MRI sequences [39–42], recent evidence suggests that multi-sequence MRI radiomics provides superior predictive performance compared to single-sequence approaches [13, 43–45]. To minimize feature redundancy and simplify dimensionality reduction, our study focused on axial and sagittal T2WI and DWI sequences for feature extraction. T2WI sequences are essential for assessing tumor size and morphology, while DWI offers valuable metabolic information. These sequences are widely recognized for radiomic feature extraction [40, 46]. Our study incorporated first-order features, texture features, and wavelet transform features to provide a comprehensive representation of tumor imaging. First-order features reflect the distribution of gray levels, while texture features—derived from GLCM, GLSZM, and GLDM—reveal the internal structure and texture of the tumor. Wavelet transform features enhance texture detail by adjusting the balance of high and low-frequency signals and using LoG features to smooth images and capture tumor heterogeneity [47]. Shape features were not included in our study due to their focus on geometric shape and structure (such as volume and shape complexity), which are less effective than texture and wavelet transform features in revealing tumor heterogeneity and intricate details.

Radiomics is primarily concerned with the morphological characteristics of tumors, providing detailed insights into tumor growth and spread. However, relying solely on radiomic features may not capture the full extent of a patient’s systemic inflammation and nutritional status—factors that play a crucial role in tumor behavior, metastasis, and treatment response. This understanding has led to a shift from traditional prognostic methods towards a more comprehensive approach that includes overall patient health. By integrating peripheral blood markers such as NLR, PLR, ELR, SII, and FNI, we can acquire a more nuanced view of a patient’s systemic health, improving the accuracy of disease progression predictions [31, 32, 48–51]. Our findings demonstrate that changes in SII before and after treatment, as well as pre-treatment FNI, are significantly associated with the prognosis of CC patients. Specifically, SII reflects the overall systemic inflammatory burden, where elevated levels often indicate a more robust inflammatory response, which correlates with tumor aggressiveness and metastatic potential. The fluctuations in SII during treatment can promptly indicate shifts in tumor burden and immune response, thus offering more accurate prognostic predictions. Similarly, pre-treatment FNI provides an effective measure of nutritional status, which directly affects immune cell function and quantity, thereby influencing immune response and overall prognosis [52].

Recent advancements in combined predictive models have markedly improved the accuracy of survival outcome predictions, treatment responses, and metastasis risk. Research has consistently shown that multi-indicator models outperform single-indicator models in terms of predictive performance. For example, Zhang et al. found that a model integrating T-stage, LNM location, and radiomic scores significantly outperformed models based solely on clinical features or radiomics [38]. Similarly, Cai et al. demonstrated that combining radiomic scores with four clinical and pathological factors improved the C-index from 0.778 to 0.821 in the training set and from 0.816 to 0.829 in the validation set, indicating enhanced predictive capability [53]. Combining radiomics with systemic inflammatory and nutritional indicators provides a more holistic view of a patient’s health than either method alone. This approach not only evaluates local tumor characteristics but also considers the patient’s overall systemic condition, offering a more comprehensive disease assessment. Moreover, incorporating multiple indicators accounts for variability among patient populations, thereby improving the accuracy and relevance of predictions. Fang et al. have shown that a combined model integrating MRI radiomics, clinical pathological features, and peripheral blood parameters offers superior predictive performance [54]. In our study, a model that integrates clinical features, SII, FNI, and radiomics demonstrated superior predictive capability compared to models using only some of these indicators. The nomogram developed from this integrated model accurately predicted 3-year and 5-year PFS rates, with AUC values of 0.941 and 0.973, respectively. Compared to Zhang et al.’s model, which was based on IVIM-DWI parameters and Rad-Score before and after treatment [28], our model achieved higher predictive accuracy with a more streamlined approach. Furthermore, it leveraged a large, multi-center patient cohort, optimizing its performance. Our findings indicate that combining radiomics with both SII and FNI provides superior predictive performance compared to using radiomics with either SII or FNI alone. This enhancement is attributed to the complementary nature of SII and FNI, which together provide a more comprehensive assessment of the patient’s overall health. Research emphasizes that inflammatory responses are crucial in the development and progression of cancer-related malnutrition, which in turn exacerbates inflammation, creating a harmful feedback loop. This cycle weakens the immune system, increases the risk of infections and complications, and negatively affects treatment outcomes and prognosis [55]. These insights highlight the importance of simultaneously monitoring both inflammatory responses and nutritional status in clinical practice.

It is crucial to note that all our models were constructed based on single-factor analysis results, which revealed the independent predictive value of each indicator for PFS. However, the increased complexity of multivariate models may have led to the attenuation of some variables’ effects. In our study, while age, SCC-Ag, SII, and FNI did not reach significance in the multivariate analysis, models incorporating these variables collectively showed superior C-index values in both the training and validation cohorts. This indicates that these indicators may provide valuable predictive insights through their interactions with other variables, highlighting the intricate roles of the tumor microenvironment, patient physiological characteristics, and systemic inflammation in disease progression. Therefore, these findings highlight the need for future research to further investigate variable selection and model-building strategies to enhance the accuracy and clinical applicability of predictive models.

While our combined model demonstrates impressive performance, several limitations must be considered. First, the retrospective nature of our study highlights the need for prospective studies to confirm our findings. Second, in this study, the DWI b-value was set at 800 s/mm². While this provided valuable information, it may have restricted the scope of the data. Using multi-b value DWI, particularly with higher b-values, could offer a more nuanced characterization of tissue properties and result in richer radiomic data. Future research in this area is warranted to explore these possibilities. Third, our analysis was based solely on features extracted from the primary tumor prior to treatment, neglecting characteristics of metastatic lymph nodes. Since metastatic lymph node features could provide significant insights into disease progression, future studies should include radiomic features from these nodes, as well as peritumoral regions and other relevant areas, to improve predictive accuracy. Finally, our research concentrated on 1-year, 3-year, and 5-year PFS. Future research should expand to include longer-term overall survival assessments and investigate how integrating radiomics with systemic inflammatory and nutritional markers can better predict long-term quality of life for patients.

## Conclusion

Our study demonstrates that integrating clinical features, systemic inflammatory markers, immune-nutritional indices, and MRI radiomics into a combined model provides an effective tool for predicting PFS in CC patients undergoing CCRT. The nomogram developed demonstrates superior performance in forecasting 1-year, 3-year, and 5-year PFS, offering enhanced accuracy. This model facilitates personalized treatment by providing precise patient risk stratification and supporting informed clinical decision-making.

## Electronic supplementary material

Below is the link to the electronic supplementary material.

Supplementary Material 1

## Data Availability

No datasets were generated or analysed during the current study.

## Abbreviations

CC: Cervical Cancer
PFS: Progression-Free Survival
CCRT: Concurrent Chemoradiotherapy
MRI: Magnetic Resonance Imaging
FIGO: International Federation of Obstetrics and Gynaecology
SII: Systemic Immune-Inflammation Index
PNI: Prognostic Nutritional Index
NLR: Neutrophil-to-Lymphocyte Ratio
PLR: Platelet-to-Lymphocyte Ratio
MLR: Monocyte-to-Lymphocyte Ratio
ELR: Eosinophil-to-Lymphocyte Ratio
HALP: Hemoglobin-Albumin-Lymphocyte-Platelet Index
WBC: White Blood Cell count
NEU: Neutrophil count
LYM: Absolute Lymphocyte count
MONO: Monocyte count
EOS: Eosinophil count
HB: Hemoglobin
Alb: Albumin
GLCM: Gray-Level Co-occurrence Matrix
GLSZM: Gray-Level Size Zone Matrix
GLRLM: Gray-Level Run Length Matrix
NGTDM: Neighboring Gray Tone Difference Matrix
GLDM: Gray-Level Dependence Matrix
LoG: Laplacian of Gaussian
LBP: Local Binary Pattern
ICC: Intraclass Correlation Coefficient
LASSO: Least Absolute Shrinkage and Selection Operator
AUC: Area Under the Curve
ROC: Receiver Operating Characteristic
DCA: Decision Curve Analysis
VOI: Volume of Interest
EBRT: External Beam Radiotherapy
ICBT: Intracavitary Brachytherapy
PACS: Picture Archiving and Communication System
SVM: Support Vector Machines
T2WI_ax: Axial T2-weighted imaging
T2WI_sag: Sagittal T2-weighted imaging
T2WI_cor: Coronal T2-weighted imaging
DWI: Diffusion-weighted imaging
ADC: Apparent diffusion coefficient
CI: Confidence Interval
