# Supplementary material for: MRI radiomics and nutritional-inflammatory biomarkers: a powerful combination for predicting progression-free survival in cervical cancer patients undergoing concurrent chemoradiotherapy
Source: Cancer Imaging. 2024 Oct 24;24:144. doi: 10.1186/s40644-024-00789-2 (PMC11515587; doi:10.1186/s40644-024-00789-2)
Supplement: Supplementary file 1 — Supplementary Material 1 [file 40644_2024_789_MOESM1_ESM.pdf]

S1 Scan parameters of each sequence in MRI

| MR Examination | TR/TE(ms) | Slice Thickness(mm) | Acquisition Matrix | FOV(mm) | Slice Gap(mm) |
|---|---|---|---|---|---|
| Axial T2WI | 4600/87 | 4.0 | 0/384/288/0 | 240×240 | 0.8 |
| Sagittal T2WI | 4810/87 | 4.0 | 384/0/0/269 | 240×240 | 0.8 |
| Sagittal T1WI | 550/18 | 4.0 | 320/0/0/240 | 240×240 | 0.8 |
| Coronal T2WI | 4850/97 | 3.5 | 0/384/307/0 | 250×250 | 0.7 |
| Axial DWI | 5940/71 | 4.0 | 112/0/0/101 | 250×250 | 0.8 |

TR, repetition time; TE, echo time; FOV, field of view

S2 Univariate and multivariate Cox regression of PFS for modeling

| | Univariate cox regression | | Multivariate cox regression | |
|---|---|---|---|---|
| | HR（95%CI） | P | HR（95%CI） | P |
| Age (<62 vs.≥62) | 0.479(0.235,1.023) | 0.0439* | 1.424(0.544, 3.731) | 0.4717 |
| Stage (I-II vs. III-IV) | 5.180(2.240,11.950) | <0.0001* | 5.603(1.304,24.079) | 0.0205* |
| SCC-Ag (<27.0 vs.≥27.0) | 3.387(1.545, 7.354) | 0.0023* | 1.136(0.404,3.198) | 0.8086 |
| LNM (Yes vs. No) | 1.030(0.690,1.470) | 0.9450 | | |
| NLR_ change (<0.4 vs.≥0.4) | 0.722(0.372, 1.404) | 0.3370 | | |
| PLR_ change (<2.1 vs.≥2.1) | 1.477(0.660,3.299) | 0.3438 | | |
| MLR_ change (<0.3 vs.≥0.3) | 1.666(0.690, 3.998) | 0.2568 | | |
| SII_ change (<2.6 vs.≥2.6) | 2.513(1.082, 5.832) | 0.0323* | 1.122(0.352,3.577) | 0.8454 |
| SIRI_ change (<3.4 vs.≥3.4) | 2.253(0.981, 5.137) | 0.0561 | | |
| ELR_ change (<0.1 vs.≥0.1) | 0.700(0.328,1.492) | 0.3557 | | |
| HALP_ pre (<30.8 vs.≥30.8) | 0.584(0.283,1.205) | 0.1471 | | |
| FNI_ pre (<44.7 vs.≥44.7) | 0.335(0.172, 0.649) | 0.0013* | 0.441(0.168,1.154) | 0.0952 |
| Imagescore | 2.378(1.833, 3.090) | <0.0001* | 2.358(1.480,3.758) | 0.0003 * |

* means P<0.05; HR=hazard ratio; CI=confidence intervals; SCC-Ag=squamous cell carcinoma antigen; pre=pre-concurrent radiochemotherapy; change=post/pre concurrent radiochemotherapy; NLR=neutrophil-to-lymphocyte ratio; PLR=platelet-to-lymphocyte ratio; MLR=monocyte to lymphocyte ratio; SII=systemic immunoinflammatory index; SIRI=systemic inflammatory response index; PNI=prognostic nutritional index; HALP=hemoglobin, albumin, lymphocyte, platelet; ELR=eosinophil-to-lymphocyte ratio

S3 The formulas concerning the Imagescore

| | Radiomics features | Coefficients |
|---|---|---|
| T2WI_ax | original_firstorder_Skewness | -0.27475103 |
| | log.sigma.1.0.mm.3D_glszm_SizeZoneNonUniformity | 0.158809995 |
| | log.sigma.2.0.mm.3D_glszm_GrayLevelNonUniformityNormalized | 0.235858337 |
| | wavelet.HLH_gldm_DependenceVariance | -0.674490404 |
| | gradient_glcm_Imc2 | -1.859034435 |
| T2WI_sag | original_firstorder_Skewness | 0.467259398 |
| | original_glcm_MaximumProbability | -0.45229266 |
| | wavelet.HLL_gldm_DependenceVariance | 1.42352297 |
| | wavelet.HHL_gldm_DependenceVariance | -0.051390042 |
| DWI | original_glcm_JointEnergy | -0.801629263 |
| | log.sigma.4.0.mm.3D_glszm_GrayLevelVariance | -0.429337447 |
| | wavelet.LHL_glszm_SizeZoneNonUniformityNormalized | -0.833044757 |
| | wavelet.LHL_gldm_LargeDependenceEmphasis | 1.051613013 |
| | wavelet.HLL_glszm_ZoneEntropy | 0.758285632 |
| | wavelet.LLL_glrlm_RunLengthNonUniformity | 1.391354734 |
